# Wearable Surface Electromyography Sensor-Based Assessment of Intermuscular Time–Frequency Coherence Following Neuromuscular Electrical Stimulation Combined with Loaded Back Squats During Tennis Forehand Strokes

**DOI:** 10.3390/s26216902

**Published:** 2026-10-31

**Authors:** Yifei Jiang, Weihao Jiang

**Affiliations:** School of Physical Education, Hangzhou Normal University, Hangzhou 311121, China; jiang_yifei0319@163.com

**Keywords:** wearable surface electromyography sensors, sensor synchronization, neuromuscular electrical stimulation, loaded back squat, tennis forehand stroke, intermuscular time–frequency coherence

## Abstract

**Objective:** This study used synchronized wearable surface electromyography sensors and high-speed video to compare post-intervention intermuscular time–frequency coherence (TFC) among three pre-competition activation conditions during the tennis forehand drive and forehand slice. **Methods:** Thirty tennis players were randomly assigned to a loaded back squat group (Group A), a low-intensity electrical stimulation plus loaded back squat group (Group B), or a neuromuscular electrical stimulation (NMES) plus loaded back squat group (Group C), with 10 participants per group. Group B received low-intensity electrical stimulation without visible muscle contractions, whereas Group C received NMES sufficient to elicit visible muscle contractions. Following the interventions, wireless surface electromyographic sensors recorded muscle signals during both stroke techniques at 2000 Hz. A high-speed video system operating at 200 Hz was synchronized with the surface electromyography system through a transistor–transistor logic trigger to identify stroke events and segment complete movement cycles. One complete stroke cycle per participant and technique was analyzed. The sensor-derived TFC area of significant coherence (AZ) was calculated for 21 predefined muscle pairs in the alpha (8–15 Hz), beta (16–30 Hz), and gamma (31–50 Hz) bands. Ordinary or Welch’s one-way ANOVA was applied according to variance homogeneity. Benjamini–Hochberg false discovery rate (FDR) correction was performed across all 63 outcomes within each stroke technique. Significant ordinary and Welch models were followed by Bonferroni and Games–Howell comparisons, respectively. **Results:** During the forehand drive, eight sensor-derived TFC outcomes remained significant after FDR correction: BB–ABS beta and gamma, BB–LD alpha, BRD–ABS alpha, GM–PM beta and gamma, VM–LD beta, and BF–ABS beta (F = 10.747–75.185, partial η^2^ = 0.443–0.848, all q ≤ 0.003). During the forehand slice, six outcomes remained significant: BB–LD alpha, BRD–ABS gamma, GM–ABS alpha, VM–ABS alpha, GLM–ABS beta, and TA–ABS gamma (F = 9.384–47.466, partial η^2^ = 0.523–0.860, all q ≤ 0.019). Group C exhibited higher post-intervention AZ values than Groups A and B for all significant outcomes. No significant A–B differences were observed during the forehand drive. During the forehand slice, Group B showed a significantly higher BRD–ABS gamma-band AZ value than Group A, whereas the other five A–B comparisons were not significant. **Conclusions:** The synchronized sensor-based assessment identified distinct post-intervention TFC patterns across muscle pairs, frequency bands, and stroke techniques under the three activation conditions. NMES combined with loaded back squats was associated with higher AZ values in selected upper-limb–trunk and lower-limb–trunk muscle pairs. However, because no pre-intervention TFC baseline or direct stroke-performance outcomes were obtained, these sensor-derived differences cannot demonstrate within-participant increases in coherence or improved tennis performance.

## 1. Introduction

Tennis is characterized by rapid changes in rally tempo, diverse stroke techniques, and frequent transitions between offensive and defensive play, placing substantial demands on players’ speed, strength, agility, coordination, and technical consistency. Athletes must rapidly adjust their movements and posture, select appropriate stroke directions, and execute sport-specific techniques under continuously changing spatial and temporal conditions [1]. The forehand stroke is fundamental to organizing attacks, controlling rally tempo, and moving the opponent around the court. Its execution requires rapid positioning, stable lower-limb support, trunk rotation, racket control, and coordinated whole-body force transmission, all of which influence a player’s ability to control rallies and manage critical points [2,3].

The forehand drive and forehand slice represent two distinct forehand techniques. The forehand drive is characterized by relatively high racket-swing velocity, large movement amplitude, and rapid force transmission, making it important during baseline attacks and sustained rallies. In contrast, the forehand slice is commonly used to vary rally tempo, control ball trajectory, manage low balls, and facilitate offensive–defensive transitions. Its execution places greater emphasis on racket-face regulation, swing-path accuracy, upper-limb control, and postural stability. These mechanical and control differences are accompanied by distinct patterns of muscular recruitment and neuromuscular coordination [4]. The two strokes therefore provide complementary motor tasks for examining technique-specific intermuscular coupling.

Pre-competition warm-up is used to improve physiological preparedness and neuromuscular readiness before sport-specific performance [5]. For tennis players, a well-structured warm-up may facilitate explosive power, rapid movement ability, and tennis-specific performance [6]. Lower-limb activation is particularly relevant because forehand strokes depend on lower-limb support and weight transfer, followed by trunk rotation and racket-side upper-limb control [7,8]. However, this functional sequence does not imply that forehand performance is determined by lower-limb activation alone; rather, it highlights the coordinated contribution of multiple body segments.

Loaded back squats were selected as the common conditioning activity in the present study because they simultaneously engage the quadriceps, gluteal muscles, hamstrings, and trunk stabilizers while providing a standardized and controllable lower-limb stimulus. When performed with an appropriate load and recovery interval, resistance-based conditioning activities may induce post-activation performance enhancement through changes in neural excitability, motor-unit recruitment, muscle temperature, and contractile readiness [9,10]. Loaded back squats may therefore provide a practical means of preparing the lower-limb neuromuscular system before subsequent tennis-specific movements.

Neuromodulatory strategies have increasingly been investigated as adjuncts to pre-exercise preparation. Centrally targeted techniques, including transcranial direct current stimulation and transcranial magnetic stimulation, may modulate neural drive and subsequent motor status [11,12]. However, their requirements for specialized equipment, controlled operating conditions, and safety procedures may limit their practical integration into routine pre-competition warm-up. Neuromuscular electrical stimulation (NMES), in contrast, directly stimulates the peripheral neuromuscular system and can be applied concurrently with conventional resistance exercise.

By eliciting contractions in selected muscles, NMES may increase local neuromuscular excitability and facilitate motor-unit recruitment [13]. When appropriate stimulation and loading parameters are used, NMES may produce acute neuromuscular facilitation and influence subsequent explosive or rapidly executed movements [14]. In the present study, NMES was applied bilaterally to the rectus femoris during loaded back squats to provide an additional peripheral stimulus during the conditioning activity. Nevertheless, previous NMES research has focused mainly on strength, jumping, rehabilitation, and general motor tasks, and its association with intermuscular coupling during tennis-specific movements remains insufficiently understood [15].

Surface electromyography can characterize the activity of individual muscles, but amplitude-based measures alone do not fully capture the shared control relationships among muscles. Intermuscular time–frequency coherence (TFC) quantifies frequency-specific coupling between electromyographic signals across the temporal course of a movement. It is therefore suitable for examining whether different pre-competition activation conditions are associated with distinct coupling patterns across selected muscles and frequency bands. Because the forehand drive and forehand slice differ in their mechanical and coordinative requirements, any post-intervention TFC differences may also vary according to stroke technique.

Given the limited tennis-specific evidence regarding NMES combined with resistance-based pre-competition activation, this study compared post-intervention intermuscular TFC among three conditions: loaded back squats alone, low-intensity electrical stimulation combined with loaded back squats, and NMES combined with loaded back squats. TFC was examined across 21 predefined muscle pairs in the alpha, beta, and gamma bands during the forehand drive and forehand slice. We hypothesized that NMES combined with loaded back squats would be associated with higher AZ values in selected muscle pairs and frequency bands than the other two conditions. We further hypothesized that the patterns of between-group differences would vary according to stroke technique, reflecting the distinct movement demands of the forehand drive and forehand slice.

## 2. Methods

### 2.1. Participants

This study employed a three-group parallel controlled design and included 30 tennis players with systematic training experience. Before participant recruitment, an a priori sample size calculation was performed using G*Power version 3.1.9.7. The test family was set to F tests, and the statistical model was specified as ANOVA: Fixed effects, omnibus, one-way. Based on the three-group parallel design and the expected intervention effect, the effect size was set at f = 0.60, the significance level at α = 0.05, the statistical power at 1 − β = 0.80, and the number of groups at three. The calculation indicated that a minimum of 30 participants was required. The assumed effect size of f = 0.60 was informed by the between-condition differences in intermuscular TFC reported by Zhuang et al. [16] during a badminton smashing task following pre-activation interventions. This study provided a relevant racket-sport context for anticipating a large effect. However, f = 0.60 was used as a planning assumption rather than a directly calculated effect-size estimate from that study. Differences in the participants, stroke tasks, and intervention conditions limit its transferability to the present investigation. Accordingly, 30 eligible tennis players were included and randomly assigned using a random-number table to the loaded back squat group (Group A), the low-intensity electrical stimulation plus loaded back squat group (Group B), or the NMES plus loaded back squat group (Group C), with 10 participants in each group.

The inclusion criteria were as follows: long-term and systematic tennis training experience; ability to perform loaded back squats, forehand drives, and forehand slices using standardized techniques; maintenance of a normal training status during the testing period; no acute sports injury within the preceding 3 months that could affect training or sport-specific testing; no neurological disorder, severe cardiovascular disease, or other condition that could affect neuromuscular function; no contraindication to NMES; and voluntary participation with written informed consent. The exclusion criteria were as follows: musculoskeletal dysfunction affecting the performance of forehand strokes; skin lesions or other conditions at the electrode placement sites that precluded electrical stimulation; marked discomfort or inability to complete the prescribed intervention; or severe loss, saturation, synchronization failure, or movement artifacts in the surface electromyographic signals that rendered the data unsuitable for subsequent analysis.

The intervention condition was treated as a between-subject factor with three levels. The loaded-squat group performed lower-limb loaded back squats alone. The intervention condition was treated as a between-subject factor with three levels. Group A performed loaded back squats alone. Group B received low-intensity electrical stimulation at or below the sensory threshold during the same loaded back squats, without visible muscle contractions. Group C received NMES sufficient to elicit visible muscle contractions during the same loaded back squats. Each participant received only the pre-competition intervention assigned to their group. Following the intervention, all participants performed forehand drive and forehand slice tests while surface electromyographic signals were synchronously recorded from the target muscles. The three groups followed identical protocols for loaded-squat intensity, number of sets and repetitions, interset recovery, post-intervention recovery, ball-feeding procedures, stroke position, testing order, high-speed video recording, and surface electromyographic data acquisition.

Before testing, all participants were fully informed of the study purpose, experimental procedures, potential risks, possible adverse reactions, and their right to withdraw. Written informed consent was obtained from each participant before participation. The study was conducted in accordance with the ethical principles of the Declaration of Helsinki, and the experimental protocol was reviewed and approved by the Ethics Committee of Hangzhou Normal University. Participant characteristics are presented in Table 1. This study was approved by the Ethics Committee of Hangzhou Normal University (Approval No. HNU202606030). The original ethics approval document has been uploaded as Supplementary Material.

### 2.2. Experimental Equipment

#### 2.2.1. Neuromuscular Electrical Stimulation Device

**Figure 1:**
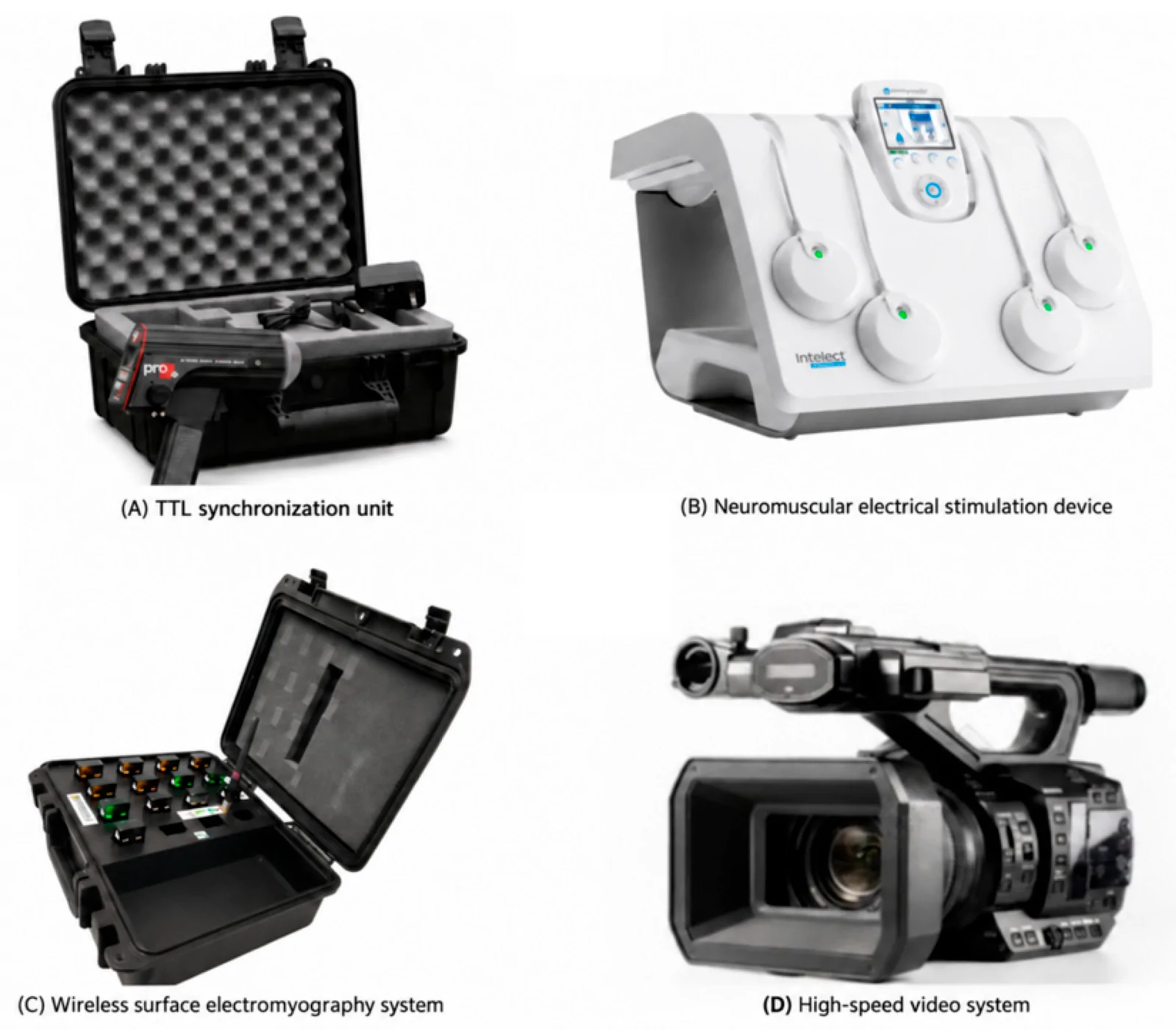
Experimental test setup.

The pre-competition NMES intervention was administered using a Compex SP 8.0 neuromuscular electrical stimulation device (Compex/DJO, Vista, CA, USA). The device delivers biphasic rectangular pulses over a stimulation frequency range of 1–120 Hz and a pulse-width range of 200–400 μs. Self-adhesive electrodes measuring 5 cm × 5 cm were used. The device was used to administer electrical stimulation in Groups B and C. Group B received low-intensity electrical stimulation at or below the sensory threshold without visible muscle contractions. Group C received NMES at an intensity sufficient to elicit visible muscle contractions while remaining tolerable to the participant. Electrode placement and device-connection procedures were identical between Groups B and C (Figure 1A).

#### 2.2.2. Surface Electromyography System

Surface electromyographic signals were recorded using a Delsys Trigno Wireless EMG System (Delsys Inc., Natick, MA, USA). This wireless multichannel surface electromyography system was configured at a sampling frequency of 2000 Hz, with an acquisition bandwidth of 20–450 Hz. The system was used to synchronously record surface electromyographic signals from the target muscles while participants performed the forehand drive and forehand slice. These recordings provided the raw data for subsequent electromyographic signal preprocessing and intermuscular time–frequency coherence analysis (Figure 1B).

#### 2.2.3. High-Speed Video System

Forehand stroke movements were recorded using a Photron FASTCAM Mini AX200 high-speed video system (Photron Limited, Tokyo, Japan) at a frame rate of 200 Hz. The camera was positioned to capture the entire forehand stroke and the instant of racket–ball contact. It continuously recorded the complete movement sequences of the forehand drive and forehand slice. The high-speed recordings were used for offline frame-by-frame identification of key movement events, including the onset of the preparation phase, maximum backswing, onset of the forward swing, racket–ball contact, and completion of the follow-through. These events were subsequently used to define the complete forehand stroke cycle and its constituent phases (Figure 1C).

#### 2.2.4. Synchronized Data Acquisition System

To establish temporal correspondence between the surface electromyographic and high-speed video recordings, the two acquisition systems were synchronized using a transistor–transistor logic (TTL) trigger. Before formal testing, the surface electromyography and high-speed video systems were connected to an external synchronization unit and configured to receive a common trigger signal. At the beginning of each recording, the synchronization unit generated a TTL pulse that provided a shared temporal reference for both systems.

Surface electromyographic signals were recorded at 2000 Hz, whereas high-speed video was recorded at 200 Hz. Although the sampling frequencies differed, both systems recorded the same physical time interval relative to the common TTL trigger. The timing of each video frame was determined from the video sampling frequency and referenced to the shared temporal origin. The beginning and end of each stroke cycle were identified frame by frame from the video, and their corresponding times were used to locate and extract the matching samples from the surface electromyographic recordings. Therefore, the video-defined movement cycle and the extracted electromyographic segment represented the same physical period of the stroke.

### 2.3. Study Design

This study employed a three-group parallel controlled design. Thirty tennis players were randomly assigned to a loaded back squat group (Group A), a low-intensity electrical stimulation plus loaded back squat group (Group B), or an NMES plus loaded back squat group (Group C), with 10 participants per group. Each participant received only the assigned intervention. Following the intervention, participants performed the forehand drive and forehand slice while surface electromyographic signals were synchronously recorded. The design was used to compare post-intervention intermuscular time–frequency coherence among the three conditions.

Loaded back squats were used as the common conditioning activity to provide a standardized lower-limb stimulus while controlling exercise-related fatigue. All groups performed loaded back squats at 60% of each participant’s one-repetition maximum (1RM), completing three sets of five repetitions with a 2 min interset recovery period. Previous conditioning research has examined three sets of five repetitions of loaded half squats at 60% 1RM [17], providing relevant precedent for the loading intensity and repetition volume used here. Exercise intensity, range of motion, movement tempo, and recovery intervals were standardized across groups. Group A performed the protocol without electrode placement or electrical stimulation. Groups B and C underwent identical electrode placement and device-connection procedures during the same squat protocol. Group B received stimulation at or below the sensory threshold without visible muscle contractions, whereas Group C received effective NMES at 75 Hz, with a pulse width of 400 μs and a duty cycle of 6 s on and 12 s off. The stimulation intensity was set at 80–90% of each participant’s maximum tolerable intensity and applied bilaterally to the rectus femoris muscles [18,19]. Group B was designated as the low-intensity electrical stimulation group because electrical current was delivered despite the absence of visible muscle contractions. This condition provided a comparison involving electrode placement, device connection, and low-intensity stimulation. It was not a no-current low-intensity electrical stimulation condition, and physiological effects of the low-intensity stimulation could not be excluded.

In this study, “low-intensity electrical stimulation” was operationally defined as electrical stimulation delivered at an amplitude that did not elicit visible muscle contractions. This criterion distinguished Group B from Group C, in which NMES elicited visible muscle contractions. Stimulation amplitude and the evoked muscular response are important considerations when characterizing electrical stimulation protocols [13]. Previous experimental research has also distinguished contraction-inducing stimulation from stimulation delivered at an amplitude insufficient to elicit muscle contraction [19]. These references support the distinction between stimulation conditions, rather than the classification of Group B as a sham control. Accordingly, Group B was designated as the low-intensity electrical stimulation plus loaded back squat group. It was not an electrode-only, no-current sham control, and the absence of visible contractions was not interpreted as evidence of physiological inactivity.

Sport-specific testing commenced within a standardized 6 min recovery window after the intervention. All tests were conducted on the same standard tennis court. A designated coach or hitting partner delivered balls using standardized trajectories, landing locations, heights, and feeding rhythms. Participants were instructed to perform the forehand drive and forehand slice at an effort approximating their usual competitive-play intensity, with the order of the two techniques randomized and balanced across groups. The court, ball-feeding procedure, intended ball trajectory, landing location, feeding height and rhythm, technical instructions, recovery intervals, and data-acquisition procedures were standardized across groups. However, actual stroke intensity and technical quality were not quantitatively assessed using ball velocity, racket velocity, stroke accuracy, returned-ball placement, or a standardized technical-quality score. The court, ball-feeding procedure, target area, technical instructions, recovery intervals, and data-acquisition procedures were kept consistent across groups.

**Figure 2:**
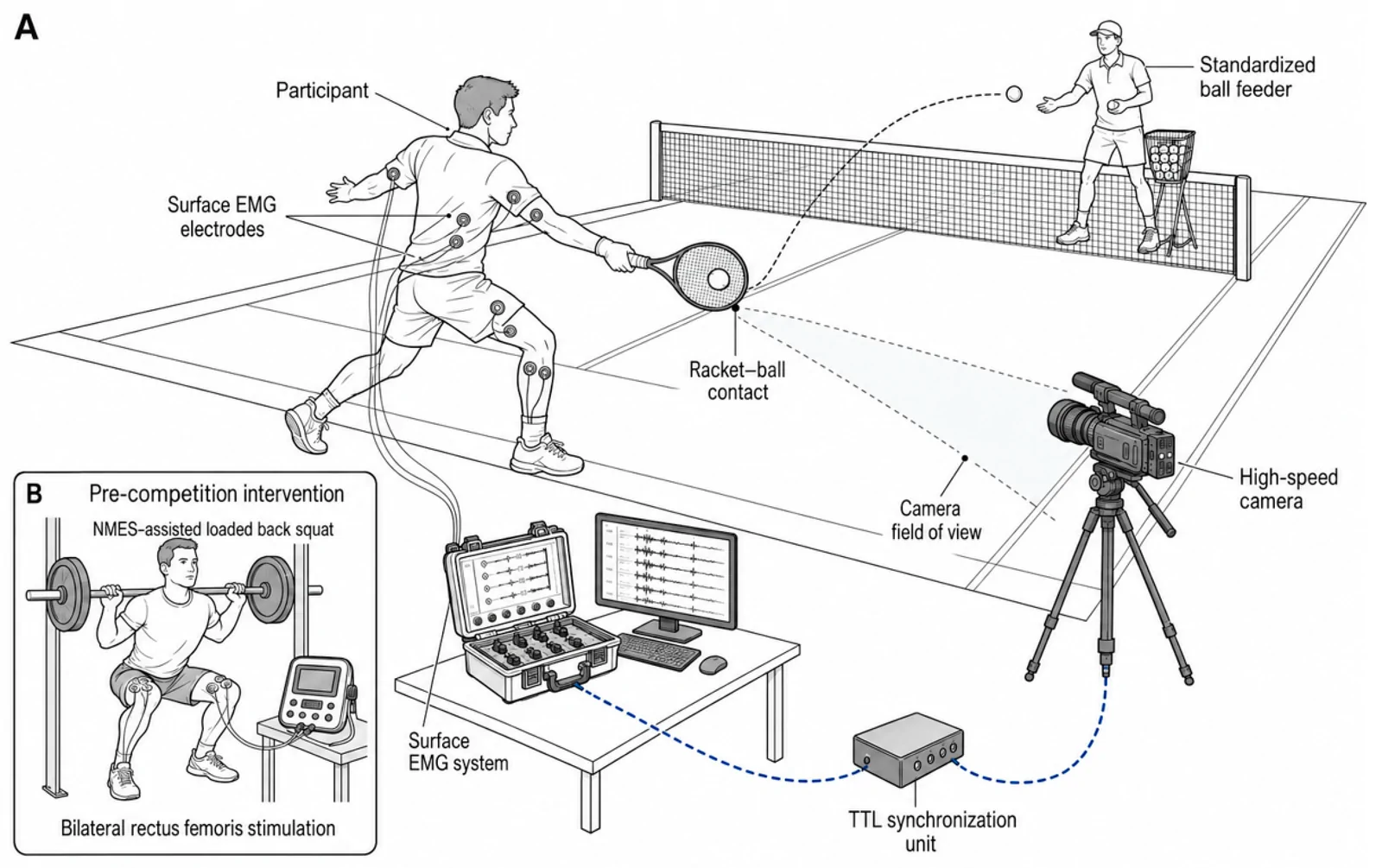
Representative schematic illustration of the actual experimental setup and sensor-placement protocol. (**A**) Tennis forehand-stroke testing with synchronized surface electromyographic and high-speed video acquisition. The surface electromyographic sensors were positioned over the predefined target muscles on the dominant racket side. (**B**) Neuromuscular electrical stimulation applied bilaterally to the rectus femoris muscles during loaded back squats. The illustration was constructed according to the actual experimental arrangement and sensor locations. A schematic representation was used to protect participant privacy and to provide a clear presentation of the sensor-placement and testing procedures. Note: (**A**) Experimental setup for tennis forehand stroke testing. Surface electromyographic signals and high-speed video recordings were synchronized using a transistor–transistor logic synchronization unit. A standardized ball feeder delivered balls according to predetermined trajectories, landing locations, heights, and feeding rhythms. (**B**) Pre-competition intervention involving neuromuscular electrical stimulation applied bilaterally to the rectus femoris muscles during loaded back squats. Surface EMG electrodes shown in Panel (**A**) were used for signal acquisition during the stroke tests and were methodologically distinct from the NMES electrodes shown in Panel (**B**).

Each participant completed 10 repetitions of each stroke technique. High-speed video and surface electromyographic data were synchronously recorded using a transistor–transistor logic trigger. For each participant and technique, one complete stroke cycle comprising the preparation, forward-swing, and follow-through phases was identified from the video recordings, and the corresponding electromyographic signals were extracted for TFC analysis. Cycle selection was based on the completeness of the recorded movement rather than stroke success or returned-ball placement. The experimental setup is shown in Figure 2.

Participants in Groups B and C underwent identical electrode-placement and device-connection procedures and were not informed of their assigned stimulation condition. However, differences in stimulation sensation and the presence of visible muscle contractions in Group C may have revealed the assigned condition; therefore, successful participant blinding could not be assumed. Group identities were coded, and the investigators responsible for identifying movement events, processing the electromyographic data, and conducting the statistical analyses remained unaware of the correspondence between group codes and intervention conditions until all analyses were completed.

### 2.4. Selection of Target Muscles

Surface electromyographic signals were recorded from muscles on the dominant racket side according to the movement characteristics of the tennis forehand drive and forehand slice and the requirements of the intermuscular time–frequency coherence analysis. The recording side was determined according to each participant’s actual racket hand before testing. For right-handed players, the relevant muscles on the right side were recorded; for left-handed players, the corresponding muscles on the left side were recorded. To ensure measurement consistency, the same recording side and electrode placement sites were maintained for each participant throughout all testing procedures. Before sensor attachment, hair was removed where necessary, and the skin was cleaned with alcohol and allowed to dry. Sensor placement followed the general surface electromyography recommendations described by Hermens et al. [20]. The target muscle bellies were identified using anatomical landmarks and palpation during muscle-specific contractions. The electrode detection axis was aligned with the local muscle-fiber direction. Placement over tendons or close to the borders of adjacent muscles was avoided where feasible to reduce the potential influence of cross-talk. Sensors were secured with adhesive fixation without restricting stroke execution. The same placement procedure was applied across participants, and sensor positions were maintained throughout both stroke tests.

The recorded muscles were categorized into trunk, upper-limb, and lower-limb groups. The trunk muscles included the rectus abdominis (ABS), latissimus dorsi (LD), and pectoralis major (PM). The upper-limb muscles included the biceps brachii (BB) and brachioradialis (BRD). The lower-limb muscles included the medial gastrocnemius (GM), vastus medialis (VM), biceps femoris (BF), gluteus maximus (GLM), and tibialis anterior (TA).

Muscle-pair selection was predefined according to the functional relationships between the upper- or lower-limb muscles and the trunk muscles during tennis forehand strokes. The seven limb muscles (BB, BRD, GM, VM, BF, GLM, and TA) were each paired with the three trunk muscles (ABS, LD, and PM), resulting in 21 predefined muscle pairs: BB–ABS, BB–LD, BB–PM, BRD–ABS, BRD–LD, BRD–PM, GM–ABS, GM–LD, GM–PM, VM–ABS, VM–LD, VM–PM, BF–ABS, BF–LD, BF–PM, GLM–ABS, GLM–LD, GLM–PM, TA–ABS, TA–LD, and TA–PM. The analysis therefore did not include all 45 mathematically possible pairwise combinations among the 10 recorded muscles [16].

The bilateral rectus femoris muscles were selected as the NMES targets because of their contribution to knee extension during loaded back squats. The stimulated muscles and the recorded muscles served different methodological purposes: NMES was used as part of the conditioning intervention, whereas surface EMG was recorded from the dominant-side limb and trunk muscles during the subsequent stroke tests. Rectus femoris activity was not recorded; therefore, the study did not directly assess activation of the stimulated muscles or a causal pathway between rectus femoris stimulation and the observed TFC outcomes.

### 2.5. Testing Procedures and Stroke-Cycle Segmentation

Following the intervention, participants commenced tennis-specific testing within a standardized 6 min window. Following the intervention, participants commenced tennis-specific testing within a standardized 6 min window. Each participant completed 10 repetitions of the forehand drive and 10 repetitions of the forehand slice, with the testing order randomized and balanced across groups. Ball-feeding procedures, technical instructions, recovery intervals, and data-acquisition procedures were standardized. High-speed video and surface electromyographic signals were recorded synchronously.

All recorded trials were initially screened according to the same prespecified eligibility criteria. A trial was considered eligible when it contained a complete movement sequence comprising the preparation, forward-swing, racket–ball contact, and follow-through phases; the key movement events could be clearly identified from the synchronized high-speed video; and the corresponding surface electromyographic recordings were complete and usable for TFC analysis. When multiple trials satisfied these criteria, one eligible trial was selected at random for each participant and stroke technique.

Trial selection was not based on stroke success, returned-ball placement, perceived technical quality, stroke intensity, coherence magnitude, group differences, or subsequent statistical results. Each participant therefore contributed one randomly selected eligible TFC dataset for each stroke technique, and repeated trials were not treated as independent statistical observations. The selected cycle was treated as the participant-level observation specified in the analytical protocol and was not assumed to represent the full range of within-participant variability across repeated trials.

For both techniques, the preparation phase extended from the onset of continuous backward racket motion to the transition into forward motion; the forward-swing phase extended from this transition to racket–ball contact; and the follow-through phase extended from immediately after contact until forward racket motion ceased and the participant began to regain balance or return to the ready position. With reference to the synchronized video–EMG approach described by Zhuang et al. [16], the stroke cycle and key movement events were identified through frame-by-frame inspection of the synchronized high-speed video. In the present study, racket–ball contact was identified using visible ball deformation, a change in ball direction, or the minimum observable racket–ball distance. The corresponding event time was then mapped to the EMG recording using the shared temporal reference.

Unsuccessful strokes were not automatically excluded or repeated if they met the predefined movement-completeness and data-quality criteria. The selected cycle was not assumed to represent the participant’s highest-intensity or best-quality performance. Video-defined cycle boundaries were mapped to the corresponding EMG samples using the shared TTL reference described in Section 2.2.4. The extracted EMG signals retained their original sampling frequency of 2000 Hz and physical duration for preprocessing and TFC estimation.

### 2.6. Data Processing and Analysis

#### 2.6.1. Surface Electromyographic Signal Preprocessing

Surface electromyographic signal preprocessing and intermuscular time–frequency coherence (TFC) analysis were performed using custom-written Python scripts with reference to the coherence-analysis framework proposed by Halliday et al. [21]. For each participant and stroke technique, preprocessing was applied to the electromyographic signals corresponding to the complete stroke cycle identified from the synchronized high-speed video recordings, as described in Section 2.5.

The raw surface electromyographic signals were band-pass filtered at 20–400 Hz using a fourth-order Butterworth filter. To attenuate 50 Hz power-line interference, a second-order infinite impulse response (IIR) notch filter with a center frequency of 50 Hz and a quality factor of 30 was applied to the signals at their original sampling frequency of 2000 Hz. The notch filter was implemented using forward–backward filtering to avoid phase distortion. Both filtering steps were performed before full-wave rectification and TFC estimation. The filtered signals were subsequently full-wave rectified.

For each participant and each stroke technique, one complete stroke cycle was extracted from the repeated trials and included in the TFC analysis. The surface electromyographic signals within the extracted cycle were processed at the original sampling frequency of 2000 Hz and retained their original physical duration throughout preprocessing and coherence estimation. The signals were not temporally stretched, compressed, or resampled to 500 points before spectral analysis. Temporal normalization to 500 points was applied only to the time dimension of the resulting time–frequency coherence map after coherence estimation.

#### 2.6.2. Intermuscular Time–Frequency Coherence Calculation

STFT coefficients were calculated at the original sampling frequency of 2000 Hz using a 200 ms Hamming window, corresponding to 400 samples. Adjacent windows overlapped by 75%, corresponding to 300 samples, with a hop size of 100 samples (50 ms). The FFT length was 400 samples, without frequency-domain zero-padding, yielding a frequency-bin spacing of 5 Hz (2000/400). Spectral estimation was performed using the original physical time base before temporal normalization of the resulting coherence maps.

For the *k*th STFT window, the complex Fourier coefficients of the two electromyographic signals were denoted by *X_k_*(*f*) and *Y_k_*(*f*), respectively. The window-specific cross-spectrum and autospectra were calculated as:

$$S_{\mathrm{xy},k}\left(f\right)=X_{k}\left(f\right)Y_{k}^{*}\left(f\right),$$

*S_xx_*_,*k*_(*f*) = |*X_k_*(*f*)|^2^, *S_yy_*_,*k*_(*f*) = |*Y_k_*(*f*)|^2^.

To obtain time-resolved spectral estimates, the cross-spectrum and autospectra at each time location were locally averaged across a centered neighborhood of nine adjacent STFT windows using equal normalized weights. At the beginning and end of the stroke cycle, the averaging neighborhood was truncated to the available windows. The locally averaged spectra were calculated as:

$${\bar{S}}_{\mathrm{xy}}\left(t,f\right)=\sum_{k\in K_{t}}w_{t,k}S_{\mathrm{xy},k}\left(f\right),$$

$${\bar{S}}_{\mathrm{xx}}\left(t,f\right)=\sum_{k\in K_{t}}w_{t,k}S_{\mathrm{xx},k}\left(f\right),{\bar{S}}_{\mathrm{yy}}\left(t,f\right)=\sum_{k\in K_{t}}w_{t,k}S_{\mathrm{yy},k}\left(f\right),$$

where 𝒦*_t_* represents the set of STFT windows contributing to the spectral estimate at time *t*, and *w_t_*_,*k*_ = 1/∣𝒦*_t_*∣. Time–frequency coherence was subsequently calculated as:

$$C_{\mathrm{xy}}\left(t,f\right)=\frac{|{\bar{S}}_{\mathrm{xy}}\left(t,f\right){|}^{2}}{{\bar{S}}_{\mathrm{xx}}\left(t,f\right){\bar{S}}_{\mathrm{yy}}\left(t,f\right)}.$$

The resulting coherence values were bounded between 0 and 1 according to the coherence definition [22]. No additional global min–max normalization was applied to the coherence maps.

#### 2.6.3. Significance Threshold and Surrogate-Data Correction

The pointwise threshold and surrogate correction were used to construct the participant-level AZ measure and were not used to support separate statistical inferences at individual time–frequency locations. The resulting time–frequency map was treated as an intermediate representation from which a scalar AZ value was extracted for each participant, muscle pair, and frequency band. Statistical testing was performed only on the participant-level AZ values, with FDR correction applied across all 63 muscle-pair × frequency-band outcomes within each stroke technique.

Because the 75%-overlapping Hamming windows were not statistically independent, the nominal number of locally averaged windows was not directly used as *L*. Instead, an overlap-adjusted effective number of segments, *L*_eff_(*t*), was calculated separately for each time location.

The normalized correlation between two Hamming windows separated by *m* hop sizes was calculated as:

$$\rho_{m}=\frac{\sum_{n=0}^{N-1-\mathrm{mH}}h\left(n\right)h\left(n+\mathrm{mH}\right)}{\sum_{n=0}^{N-1}h^{2}\left(n\right)},$$

where *h*(*n*) represents the Hamming-window coefficient, *N* = 400 represents the window length, and *H* = 100 represents the hop size. The correlation was set to zero when *mH* ≥ *N*, because the corresponding windows no longer overlapped.

For the normalized local averaging weights *w_t_*_,*k*_, the overlap-adjusted effective number of segments was calculated as:

$$L_{\mathrm{eff}}\left(t\right)={\left[\sum_{i\in K_{t}}\sum_{j\in K_{t}}w_{t,i}w_{t,j}\rho_{|i-j|}^{2}\right]}^{-1}.$$

This calculation accounted for both the number of windows included in the local spectral average and the statistical dependence introduced by the 75% overlap. Because fewer windows were available near the beginning and end of the stroke cycle, *L*_eff_(*t*) was calculated separately for each time location.

The pointwise 95% coherence confidence limit was then calculated as:

$$\mathrm{CL}\left(t\right)=1-\alpha^{1/\left[L_{\mathrm{eff}}\left(t\right)-1\right]},$$

where *α* = 0.05. A time–frequency location was considered statistically significant only when its observed coherence exceeded the corresponding time-specific confidence limit.

To further reduce the influence of chance coupling and the intrinsic spectral characteristics of the electromyographic signals, phase-randomized surrogate data were used. For each participant and muscle pair, 200 independent surrogate realizations were generated. The Fourier amplitudes of the original signals were retained, whereas their phases were independently randomized while preserving the conjugate symmetry required to reconstruct real-valued signals. Each surrogate realization was processed using the same STFT, local spectral-averaging, overlap-correction, and coherence-estimation procedures as the original signals.

The mean surrogate coherence map was calculated across the 200 realizations. Surrogate-corrected coherence was then calculated as:

$$C_{\mathrm{corr}}\left(t,f\right)=\max\left[C_{\mathrm{xy}},\left(t,f\right)-{\bar{C}}_{\mathrm{surr}}\left(t,f\right)0\right],$$

where *C_xy_*(*t*,*f*) represents the observed coherence and ${\bar{C}}_{\mathrm{surr}}\left(t,f\right)$ represents the mean surrogate coherence. The corrected coherence was set to zero when the observed coherence did not exceed the corresponding time-specific 95% confidence limit.

#### 2.6.4. Area of Significant Coherence

After pointwise significance thresholding and phase-randomized surrogate correction, the temporal dimension of each surrogate-corrected TFC map was linearly normalized to 500 equally spaced points representing 0–100% of the complete stroke cycle. Temporal normalization was applied to the calculated coherence map rather than to the surface electromyographic signals used for spectral estimation. The frequency axis was not normalized or rescaled.

For each predefined muscle pair and frequency band, the area of significant coherence (AZ) was calculated from the temporally normalized, surrogate-corrected TFC map as:

$$AZ_{b}=\sum_{\left(i,k\right)\in \Omega_{b}}C_{\mathrm{corrected}}\left(i,k\right)\Delta t\Delta f$$

where *b* denotes the specified frequency band; *C*_corrected_(*i*,*k*) represents the surrogate-corrected coherence at normalized time point *i* and frequency bin *k*; and *Ω_b_* represents the set of time–frequency locations within frequency band *b* at which the observed coherence exceeded the corresponding time-specific 95% confidence limit and the surrogate-corrected coherence was greater than zero.

Because the normalized temporal axis contained 500 equally spaced points including both 0% and 100% of the stroke cycle, Δ*t* was defined as 1/499 of the normalized stroke cycle, equivalent to 100/499 percentage points. The frequency interval Δ*f* was determined by the frequency resolution of the STFT and retained its physical unit of hertz. A larger AZ value therefore indicated a greater combined extent and magnitude of significant surrogate-corrected intermuscular coherence within the corresponding frequency band.

Fisher Z transformation was not applied to the pointwise coherence values or the resulting AZ values. For each participant, one AZ value was obtained for every predefined muscle-pair × frequency-band outcome during each stroke technique, and these participant-level AZ values were entered directly into the statistical analyses.

#### 2.6.5. Frequency-Band Classification and Interpretation

The selection of the frequency ranges was informed by the racket-sport TFC study of Zhuang et al. [16]. In the present analysis, the bands were defined as alpha (8–15 Hz), beta (16–30 Hz), and gamma (31–50 Hz), with both endpoints included. Frequency bins were selected according to their actual STFT frequencies. With a frequency-bin spacing of 5 Hz, the included bins were 10 and 15 Hz for alpha; 20, 25, and 30 Hz for beta; and 35, 40, 45, and 50 Hz for gamma. Thus, the boundary bins were not duplicated across bands. These definitions were applied consistently to all participants, muscle pairs, and stroke techniques. Alpha-band intermuscular coherence has been linked to the temporal organization of muscle synergies during rhythmic upper-limb movement. Beta- and gamma-band intermuscular coherence can vary across muscles and movement phases during dynamic tasks [23,24]. Related corticomuscular research has also reported task-dependent differences between beta- and gamma-range coupling during isometric and isotonic contractions [25].

These frequency-band interpretations were used to characterize possible differences in shared neural drive and intermuscular coupling. Because surface intermuscular coherence cannot directly identify the anatomical origin of common neural input, changes in a specific frequency band were not interpreted as direct evidence of increased or decreased activity within a particular central nervous system structure.

#### 2.6.6. Interpretation of the Temporal Axis

On the normalized temporal axis, 0% represented the onset of continuous backward racket motion and 100% represented completion of the post-impact follow-through. Key movement events, including the transition into the forward swing and racket–ball contact, were mapped onto this axis. Temporal normalization and AZ calculation are described in Section 2.6.4.

### 2.7. Statistical Analysis

All statistical analyses were performed using SPSS version 26.0 and independently verified using Python 3.12 with the SciPy statistical package. Continuous variables are presented as mean ± standard deviation (M ± SD). The participant was the independent unit of analysis. For each participant and stroke technique, one randomly selected eligible cycle contributed one AZ value for each predefined muscle-pair × frequency-band outcome. AZ values were analyzed without Fisher Z transformation. Individual trials and time–frequency locations were not treated as independent observations.

The study employed a three-group parallel controlled design consisting of the loaded back squat group, the low-intensity electrical stimulation plus loaded back squat group, and the NMES plus loaded back squat group, with 10 independent participants in each group. Model assumptions were evaluated separately for every muscle-pair × frequency-band outcome. The normality of model residuals was assessed using the Shapiro–Wilk test and examined using residual histograms and normal Q–Q plots. Homogeneity of variance was assessed using the mean-centered Levene test and residual-versus-fitted plots.

When the homogeneity-of-variance assumption was satisfied (Levene’s *p* ≥ 0.05), between-group differences were examined using ordinary one-way between-subjects analysis of variance (ANOVA). The results were reported as F(2,27), raw *p* values, and partial eta-squared values (partial η^2^). When the homogeneity-of-variance assumption was violated (Levene’s *p* < 0.05), Welch’s one-way ANOVA was used. For Welch’s ANOVA, the denominator degrees of freedom were estimated using the Welch–Satterthwaite approximation and could therefore be non-integer. Welch results were reported as Welch F(df_1_, df_2_), raw *p* values, and F-based partial η^2^ values. The effect sizes were calculated using the F statistic and corresponding degrees of freedom from the same statistical model, thereby ensuring internal consistency among the reported F statistic, degrees of freedom, *p* value, and effect size.

Post hoc pairwise comparisons were performed only when an omnibus group effect remained statistically significant after false discovery rate correction. For significant outcomes analyzed using ordinary ANOVA, pairwise independent-samples comparisons were conducted with Bonferroni adjustment. For significant outcomes analyzed using Welch’s ANOVA, Games–Howell pairwise comparisons were used because this procedure does not assume equal group variances. Adjusted pairwise *p* values, between-group mean differences, and 95% confidence intervals were reported. No outcome required a nonparametric analysis based on the residual normality assessment.

Because 21 predefined muscle pairs were examined in three frequency bands, each stroke technique included 63 omnibus muscle-pair × frequency-band tests. To control multiplicity across muscle pairs and frequency bands, all 63 omnibus *p* values within each stroke technique were treated as a single correction family and adjusted together using the Benjamini–Hochberg false discovery rate (FDR) procedure. Separate 63-test FDR families were defined for the forehand drive and forehand slice because they represented two prespecified stroke-specific analyses. Both the raw *p* value and corresponding FDR-adjusted q value were reported for every outcome.

All statistical tests were two-sided. An FDR-adjusted q value < 0.05 was considered statistically significant. Results with a raw *p* value < 0.05 but an FDR-adjusted q value ≥ 0.05 were not interpreted as statistically significant after multiplicity correction. Partial η^2^ values of 0.01, 0.06, and 0.14 were interpreted as small, moderate, and large effect sizes, respectively. Exact *p* and q values were reported whenever possible; values below 0.001 were reported as *p* < 0.001 and q < 0.001, respectively.

## 3. Results

For each stroke technique, the analyzed dataset comprised 30 independent participants, with 10 participants in each group. Each participant contributed one AZ value per muscle-pair × frequency-band outcome. Group A performed loaded back squats alone; Group B received low-intensity electrical stimulation combined with loaded back squats; and Group C received NMES combined with loaded back squats. The omnibus *p* values in Table 2 and Table 3 test the overall null hypothesis of equal mean AZ values across Groups A, B, and C; they do not identify which individual groups differ. The corresponding q values reflect Benjamini–Hochberg FDR correction across the 63 omnibus tests within each stroke technique. Specific between-group differences were evaluated using multiplicity-adjusted pairwise comparisons, reported in Table 4 and Table 5.

### 3.1. Intermuscular Time–Frequency Coherence During the Forehand Drive

Eight AZ outcomes across six muscle pairs showed significant overall differences among the three groups after FDR correction (Table 2). All eight outcomes satisfied the homogeneity-of-variance assumption and were analyzed using ordinary one-way ANOVA.

For upper-limb–trunk muscle pairs, significant omnibus effects were observed for BB–ABS in the beta band, F(2,27) = 10.747, raw *p* < 0.001, partial η^2^ = 0.443, q = 0.003; BB–ABS in the gamma band, F(2,27) = 13.231, raw *p* < 0.001, partial η^2^ = 0.495, q = 0.001; BB–LD in the alpha band, F(2,27) = 11.699, raw *p* < 0.001, partial η^2^ = 0.464, q = 0.002; and BRD–ABS in the alpha band, F(2,27) = 14.834, raw *p* < 0.001, partial η^2^ = 0.524, q < 0.001.

For lower-limb–trunk muscle pairs, significant omnibus effects were identified for GM–PM in the beta band, F(2,27) = 18.192, raw *p* < 0.001, partial η^2^ = 0.574, q < 0.001; GM–PM in the gamma band, F(2,27) = 34.399, raw *p* < 0.001, partial η^2^ = 0.718, q < 0.001; VM–LD in the beta band, F(2,27) = 75.185, raw *p* < 0.001, partial η^2^ = 0.848, q < 0.001; and BF–ABS in the beta band, F(2,27) = 13.129, raw *p* < 0.001, partial η^2^ = 0.493, q = 0.001. None of the remaining omnibus outcomes remained significant after FDR correction (all q ≥ 0.05).

Bonferroni-adjusted pairwise comparisons identified significantly higher AZ values in Group C than in both Groups A and B for all eight outcomes (Table 4). For C versus A, adjusted *p* values ranged from 2.15 × 10^−9^ to 0.002587; for C versus B, they ranged from 2.39 × 10^−8^ to 0.048855. No A–B comparison was significant after Bonferroni adjustment: adjusted *p* = 1.000 for seven outcomes and adjusted *p* = 0.061396 for BF–ABS in the beta band.

### 3.2. Intermuscular Time–Frequency Coherence During the Forehand Slice

Six AZ outcomes showed significant overall differences among the three groups after FDR correction (Table 3). Ordinary one-way ANOVA was used for BB–LD in the alpha band, F(2,27) = 41.421, raw *p* < 0.001, partial η^2^ = 0.754, q < 0.001, and GLM–ABS in the beta band, F(2,27) = 25.667, raw *p* < 0.001, partial η^2^ = 0.655, q < 0.001.

Welch’s ANOVA was used for the four outcomes showing variance heterogeneity: BRD–ABS in the gamma band, F(2,16.90) = 30.160, raw *p* < 0.001, partial η^2^ = 0.781, q < 0.001; GM–ABS in the alpha band, F(2,15.42) = 47.466, raw *p* < 0.001, partial η^2^ = 0.860, q < 0.001; VM–ABS in the alpha band, F(2,16.43) = 19.618, raw *p* < 0.001, partial η^2^ = 0.705, q < 0.001; and TA–ABS in the gamma band, F(2,17.09) = 9.384, raw *p* = 0.002, partial η^2^ = 0.523, q = 0.019. None of the remaining omnibus outcomes remained significant after FDR correction (all q ≥ 0.05).

Bonferroni-adjusted pairwise comparisons were used for the two ordinary-ANOVA outcomes, and Games–Howell comparisons were used for the four Welch-ANOVA outcomes (Table 5). Group C had significantly higher AZ values than both Groups A and B for all six outcomes. Adjusted *p* values ranged from 3.73 × 10^−7^ to 0.002010 for C versus A and from 1.56 × 10^−6^ to 0.004529 for C versus B.

For BRD–ABS in the gamma band, Group B also had a significantly higher AZ value than Group A (Games–Howell adjusted *p* = 0.003350). Together with the significant C–A and C–B comparisons, this supported an ordered pattern of Group C > Group B > Group A for this outcome. The other five A–B comparisons were not significant (adjusted *p* = 0.100375–1.000).

Across the two stroke techniques, 14 of the 126 prespecified muscle-pair × frequency-band outcomes showed significant post-intervention between-group differences after FDR correction, comprising eight outcomes during the forehand drive and six during the forehand slice. Group C exhibited higher AZ values than Groups A and B for all 14 outcomes.

During the forehand drive, the largest between-group effect was observed for VM–LD in the beta band (partial η^2^ = 0.848), followed by GM–PM in the gamma band (partial η^2^ = 0.718). The remaining significant outcomes involved BB–ABS in the beta and gamma bands, BB–LD and BRD–ABS in the alpha band, GM–PM in the beta band, and BF–ABS in the beta band. Thus, the significant forehand-drive outcomes were distributed across both upper-limb–trunk and lower-limb–trunk muscle pairs.

During the forehand slice, the largest between-group effect was observed for GM–ABS in the alpha band (partial η^2^ = 0.860), followed by BRD–ABS in the gamma band (partial η^2^ = 0.781), BB–LD in the alpha band (partial η^2^ = 0.754), VM–ABS in the alpha band (partial η^2^ = 0.705), GLM–ABS in the beta band (partial η^2^ = 0.655), and TA–ABS in the gamma band (partial η^2^ = 0.523). Four of the six significant slice outcomes involved lower-limb muscles paired with the rectus abdominis, indicating that the principal post-intervention group differences during this technique were concentrated in selected lower-limb–abdominal interactions.

Because TFC was assessed only after the interventions, these effect-size patterns identify the outcomes showing the greatest post-intervention separation among the three conditions; they do not establish which muscle pairs were physiologically most responsive to NMES relative to their pre-intervention values.

## 4. Discussion

The present study compared post-intervention intermuscular time–frequency coherence among the loaded back squat, low-intensity electrical stimulation plus loaded back squat, and NMES plus loaded back squat groups during the tennis forehand drive and forehand slice. After Benjamini–Hochberg false discovery rate correction across all 63 muscle-pair × frequency-band outcomes within each stroke technique, eight outcomes during the forehand drive and six during the forehand slice showed significant between-group differences. For all 14 FDR-significant outcomes, the NMES plus loaded back squat group exhibited higher AZ values than both comparison groups. No A–B differences were identified during the forehand drive, whereas the BRD–ABS gamma-band outcome during the forehand slice showed an ordered pattern of Group C > Group B > Group A. Because TFC was measured only after the interventions, these findings represent post-intervention between-group differences and cannot demonstrate within-participant changes from baseline.

Previous research has identified muscular and neural contributions to postactivation potentiation [20]. Although NMES was applied bilaterally to the rectus femoris during loaded back squats, rectus femoris EMG was not recorded during the intervention or subsequent stroke tests. Therefore, the study could not directly assess the activity of the stimulated muscles or establish a mechanistic pathway linking rectus femoris stimulation to the observed TFC differences. These findings should be interpreted as post-intervention differences in coupling between the recorded muscle signals, rather than evidence of a demonstrated mechanism originating from the stimulated rectus femoris.

During the forehand drive, significant post-intervention differences were identified in the beta- and gamma-band AZ values of BB–ABS, the alpha-band AZ values of BB–LD and BRD–ABS, the beta- and gamma-band AZ values of GM–PM, and the beta-band AZ values of VM–LD and BF–ABS. Group C exhibited higher AZ values than Groups A and B for all eight outcomes, whereas no significant A–B differences were identified. The significant outcomes involved both upper-limb–trunk coupling (BB–ABS, BB–LD, and BRD–ABS) and lower-limb–trunk coupling (GM–PM, VM–LD, and BF–ABS), indicating that the between-group differences were distributed across selected functional regions rather than confined to a single anatomical level. Nevertheless, without direct kinematic or kinetic measurements, these differences cannot be interpreted as evidence of improved force transmission or more effective kinetic-chain coordination.

Beta-band intermuscular coherence has commonly been associated with common neural modulation involving the corticospinal system, whereas gamma-band coherence may be more evident during rapid and dynamic muscle contractions [24,25]. Group C exhibited higher beta-band AZ values for BB–ABS, GM–PM, VM–LD, and BF–ABS and higher gamma-band AZ values for BB–ABS and GM–PM during the forehand drive. These findings indicate stronger frequency-specific statistical coupling between the corresponding muscle signals under the real-stimulation condition. However, surface intermuscular coherence cannot determine whether these differences originated from cortical, subcortical, corticospinal, spinal, peripheral, or mechanical sources. The beta- and gamma-band findings should therefore be interpreted as frequency-specific associations rather than direct evidence of enhanced corticospinal activity or increased common neural drive.

Group C also exhibited higher alpha-band AZ values for BB–LD and BRD–ABS than both comparison groups during the forehand drive. Alpha-band intermuscular coherence has been associated with low-frequency common input related to postural regulation and the temporal organization of muscle activity [26]. The observed differences may therefore reflect distinct post-intervention patterns of low-frequency coupling between muscles involved in racket-side upper-limb control and trunk-related functions. Nevertheless, this interpretation remains inferential because surface electromyography cannot identify the anatomical origin of the shared input or determine whether higher AZ values represent more effective postural control or superior technical coordination.

The forehand-drive findings were restricted to eight of the 63 tested muscle-pair × frequency-band outcomes. This selectivity is consistent with evidence that intermuscular coherence depends on the specific muscles, frequency ranges, and motor tasks examined [27]. Thus, the findings indicate localized rather than generalized differences in post-intervention TFC. Furthermore, because no pre-intervention TFC baseline was available, the higher AZ values in Group C cannot demonstrate that coherence increased within this group after the intervention.

During the forehand slice, significant post-intervention differences were identified in the alpha-band AZ value of BB–LD, the gamma-band AZ value of BRD–ABS, the alpha-band AZ values of GM–ABS and VM–ABS, the beta-band AZ value of GLM–ABS, and the gamma-band AZ value of TA–ABS. Group C exhibited higher AZ values than Groups A and B for all six outcomes. In addition, Group B exhibited a higher BRD–ABS gamma-band AZ value than Group A, resulting in an ordered pattern of Group C > Group B > Group A. No significant A–B differences were identified for the other five outcomes.

The forehand slice is performed with a relatively open racket face and a downward-and-forward racket trajectory, placing substantial demands on swing-path regulation, racket-face control, upper-limb deceleration, and postural adjustment [28]. The significant slice outcomes included upper-limb–trunk coupling (BB–LD and BRD–ABS) and lower-limb–trunk coupling (GM–ABS, VM–ABS, GLM–ABS, and TA–ABS). Compared with the forehand-drive findings, these outcomes involved a different combination of muscle pairs and frequency bands, supporting the task-specific nature of intermuscular coherence. However, their functional significance cannot be established without concurrent measurements of racket motion, ball performance, joint kinematics, or external forces.

The ordered Group C > Group B > Group A pattern for gamma-band BRD–ABS during the forehand slice warrants cautious interpretation. Group B received low-intensity electrical stimulation without visible muscle contractions, and the absence of visible contractions does not establish physiological inactivity. The difference between Groups A and B could therefore reflect effects of low-intensity stimulation, electrode placement, expectations, attention, or a combination of these factors. The present design cannot distinguish among these explanations. This isolated finding does not establish a generalized effect of low-intensity electrical stimulation on intermuscular coherence.

The distribution of significant outcomes differed between the two stroke techniques. The forehand drive yielded eight significant outcomes and the forehand slice yielded six, with different muscle-pair × frequency-band combinations observed for each technique. These findings suggest that the post-intervention TFC patterns varied according to the specific motor demands of the subsequent stroke. However, the number or distribution of significant outcomes should not be interpreted as evidence that one technique benefited more from the intervention than the other, because no direct statistical comparison between the two stroke techniques was performed.

The pairwise comparisons between Groups A and B were predominantly nonsignificant. Specifically, no A–B differences were observed for the eight significant forehand-drive outcomes or for five of the six significant forehand-slice outcomes. No statistically significant A–B differences were observed for the eight FDR-significant forehand-drive outcomes or for five of the six FDR-significant forehand-slice outcomes. However, these nonsignificant comparisons do not establish equivalence between the conditions or physiological inactivity of low-intensity stimulation. The significant A–B difference in gamma-band BRD–ABS during the forehand slice further indicates that Group B cannot be assumed to function as an inert control. Because the study did not include an electrode-only, no-current group, effects of low-intensity stimulation could not be separated from electrode-placement and procedural effects. Differences in stimulation sensation and visible contractions between Groups B and C also mean that expectancy effects cannot be excluded.

When the significant outcomes are considered according to their effect sizes and functional distribution, different muscle-interaction patterns are apparent between the two stroke techniques. During the forehand drive, VM–LD beta-band and GM–PM gamma-band coupling showed the largest between-group effects. The vastus medialis and medial gastrocnemius contribute to knee control, lower-limb support, and regulation of the base of support, whereas the latissimus dorsi and pectoralis major contribute to trunk–upper-limb integration during the forward swing. The significant VM–LD and GM–PM outcomes may therefore reflect differences in the coupling of lower-limb support with trunk and upper-body control during a rapidly executed forehand drive. The additional BB–ABS, BB–LD, BRD–ABS, and BF–ABS findings indicate that the observed group differences were distributed across selected upper-limb–trunk and lower-limb–trunk interactions.

During the forehand slice, the largest effect was observed for GM–ABS alpha-band coupling. Three additional significant outcomes—VM–ABS in the alpha band, GLM–ABS in the beta band, and TA–ABS in the gamma band—also involved lower-limb muscles paired with the rectus abdominis. This concentration of significant outcomes may be functionally related to the requirement for lower-limb support and trunk stabilization while regulating the downward-and-forward racket trajectory. The significant BB–LD alpha-band and BRD–ABS gamma-band outcomes may additionally reflect the coordination of racket-side upper-limb control with trunk musculature during racket-face regulation, forward movement, and deceleration.

The frequency-specific findings also require cautious interpretation. Alpha-band coherence has been associated with low-frequency common input involved in postural regulation and temporal organization, beta-band coherence with common neural modulation involving the corticospinal system, and gamma-band coherence with rapid and dynamic muscle contractions. Accordingly, the alpha-band lower-limb–abdominal findings during the slice may be consistent with its postural-control demands, whereas the beta- and gamma-band findings during the drive may be related to its more rapid and forceful execution. However, these interpretations do not identify the anatomical source of the shared input and do not demonstrate that higher coherence represents more effective coordination.

Moreover, the present findings cannot establish a causal pathway from rectus femoris stimulation to the observed muscle-pair differences. The rectus femoris was stimulated during the conditioning activity but was not recorded during the subsequent stroke tests. No pre-intervention TFC, joint kinematics, external forces, racket velocity, ball velocity, or stroke-accuracy outcomes were obtained. Therefore, the identified muscle-pair patterns should be interpreted as post-intervention statistical coupling associated with the three experimental conditions rather than direct evidence that NMES enhanced kinetic-chain transmission or tennis performance.

From a practical perspective, effective NMES combined with loaded back squats was associated with higher post-intervention AZ values in selected muscle-pair × frequency-band outcomes. Higher TFC/AZ indicates a greater extent and/or magnitude of significant frequency-specific coupling between the recorded muscle signals. However, stronger coupling does not necessarily indicate improved neuromuscular coordination or better motor performance. It may also reflect increased stabilization demands, co-contraction, compensatory control, or other neural and mechanical influences. These possible explanations were not directly assessed in the present study. Because ball velocity, racket velocity, stroke accuracy, kinetic variables, and three-dimensional kinematics were not measured, the functional benefit of the observed differences cannot be determined. Therefore, the findings should be interpreted as differences in post-intervention intermuscular coupling patterns among the three activation conditions. Further studies incorporating direct performance and biomechanical outcomes are required to determine whether these coupling patterns are associated with beneficial changes in stroke execution. The sample size calculation assumed a relatively large effect size informed by previous racket-sport research. The study may therefore have had limited power to detect smaller effects, particularly after correction for multiple comparisons.

The present study provides evidence that post-intervention intermuscular coupling patterns during tennis-specific movements differ across pre-competition activation conditions. The differences were confined to selected muscle pairs and frequency bands, and the significant outcomes differed between the forehand drive and forehand slice. These findings suggest that the association between the activation condition and subsequent muscle coupling is specific to the muscle interaction, frequency band, and stroke technique. The contribution of this study is therefore the identification of these specific coupling patterns, rather than evidence of a uniform increase in coherence across all muscle interactions.

From a practical perspective, wearable surface EMG synchronized with high-speed video provides a means of assessing intermuscular coupling within a clearly defined tennis stroke cycle. This approach may complement conventional performance assessments by characterizing relationships between muscle signals that are not captured by an isolated measure of muscle activation. The muscle pairs and frequency bands showing significant differences in this study provide candidate outcomes for future evaluations of NMES-assisted preparation strategies and tennis-specific neuromuscular assessment.

However, higher TFC or AZ should not be interpreted as evidence of improved coordination, stroke effectiveness, or competitive performance. Because the present study did not measure stroke performance or pre-intervention TFC, its findings establish post-intervention differences between groups rather than within-participant improvements or a performance benefit. Further studies combining repeated-trial coherence analysis with stroke velocity, accuracy, and other functional outcomes are needed to determine whether these coupling differences have practical benefits and whether NMES-assisted preparation can inform training or competition routines.

### Limitations of the Study

This study has several limitations. First, the small sample of trained tennis players limits generalizability. Second, the randomized three-group post-test design allowed between-group comparisons, but the absence of pre-intervention TFC measurements prevented assessment of within-participant changes and adjustment for baseline TFC differences. Third, only one randomly selected eligible stroke cycle per participant and technique was analyzed from 10 repetitions. Although random selection reduced subjective trial selection, it could not characterize within-participant variability; trial-to-trial reliability and sensitivity to trial selection or aggregation were not evaluated. Fourth, EMG was recorded only from selected muscles on the dominant racket side, excluding the bilaterally stimulated rectus femoris muscles. Consequently, a direct mechanistic link between stimulation and TFC differences, bilateral contributions, and the sources of coupling could not be established. Fifth, despite standardized testing and ball feeding, actual stroke intensity and technical quality were not quantified. The absence of ball and racket velocity, accuracy, returned-ball placement, technical-quality scores, kinetics, and three-dimensional kinematics limited assessment of stroke comparability and the functional significance of higher AZ values. Finally, pointwise significance thresholding and surrogate correction were applied without maximum-statistic or cluster-based correction across the time–frequency plane, leaving isolated false-positive locations possible. Future research should incorporate larger samples, pre–post and multiple-cycle assessments, bilateral and stimulated-muscle recordings, performance and biomechanical measures, and map-level correction. An additional limitation is that Group B received low-intensity electrical stimulation rather than an electrode-only, no-current low-intensity electrical stimulation intervention. Physiological effects of this stimulation cannot be excluded, and the design cannot separate its effects from electrode-placement, procedural, or expectancy effects. Differences in stimulation sensation and visible muscle contractions also limited participant blinding.

## 5. Conclusions

The three pre-competition activation conditions were associated with distinct post-intervention intermuscular TFC patterns during the forehand drive and forehand slice. Effective NMES combined with loaded back squats produced higher AZ values than both comparison conditions in eight forehand-drive outcomes and six forehand-slice outcomes, with different muscle-pair × frequency-band patterns observed between the two stroke techniques. The loaded back squat and low-intensity electrical stimulation plus loaded back squat groups did not differ in the significant forehand-drive outcomes or in five of the six significant forehand-slice outcomes; the only exception was gamma-band BRD–ABS during the forehand slice, which showed an ordered pattern of NMES plus loaded back squat > low-intensity electrical stimulation plus loaded back squat > loaded back squat.

These findings indicate differences in post-intervention intermuscular coupling patterns in selected muscle pairs and frequency bands. Higher AZ values do not necessarily represent improved neuromuscular coordination or better tennis performance, because stronger coupling may also reflect stabilization demands, co-contraction, compensatory control, or other neural and mechanical influences. The absence of pre-intervention TFC measurements prevents determination of within-participant changes, while the absence of direct performance and biomechanical outcomes prevents assessment of the functional benefit of the observed group differences. Further research incorporating pre–post measurements, repeated-cycle analysis, stimulated-muscle recordings, and direct performance and biomechanical assessments is required to establish their functional significance.

## Figures and Tables

**Table 1:** Basic characteristics of the participants and between-group comparisons.

Variable	Loaded Back Squat Group (*n* = 10)	Low-Intensity Electrical Stimulation + Loaded Back Squat Group (*n* = 10)	NMES + Loaded Back Squat Group (*n* = 10)	Statistic	*p* Value
Sex (male/female)	7/3	5/5	6/4	Fisher’s exact test	0.888
Age (years)	21.04 ± 1.13	21.37 ± 1.06	21.61 ± 0.96	F = 0.74	0.486
Height (cm)	178.53 ± 6.42	176.87 ± 7.91	174.85 ± 5.75	F = 0.75	0.484
Body mass (kg)	70.71 ± 6.95	70.49 ± 6.91	69.10 ± 4.65	F = 0.19	0.826
Body mass index (kg/m^2^)	22.14 ± 1.20	22.49 ± 1.00	22.60 ± 1.11	F = 0.49	0.619
Training experience (years)	7.46 ± 1.49	7.59 ± 0.70	7.54 ± 1.78	F = 0.02	0.979
Athletic classification (Level I/Master)	8/2	6/4	7/3	Fisher’s exact test	0.881
Dominant racket side (right/left)	9/1	8/2	10/0	Fisher’s exact test	0.749

Note: Continuous variables are presented as mean ± standard deviation (M ± SD) and were compared among the three groups using one-way analysis of variance, with degrees of freedom reported as F(2,27). Categorical variables are presented as numbers of participants and were compared using Fisher’s exact test. No statistically significant differences in baseline characteristics were observed among the three groups (*p* > 0.05), indicating that the groups were comparable at baseline. Group A performed loaded back squats alone; Group B received low-intensity electrical stimulation without visible muscle contractions during the same squat protocol; and Group C received NMES sufficient to elicit visible muscle contractions during the same squat protocol.

**Table 2:** Between-group comparisons of intermuscular time–frequency coherence during the forehand drive.

Muscle Pair	Frequency Band	Group A	Group B	Group C	F(2,27)	Raw *p*	Partial η^2^	FDR q
BB-ABS	β	0.360 ± 0.149	0.390 ± 0.118	0.620 ± 0.143	10.747	<0.001	0.443	0.003
BB-ABS	γ	0.370 ± 0.177	0.410 ± 0.161	0.710 ± 0.145	13.231	<0.001	0.495	0.001
BB-LD	α	0.277 ± 0.102	0.306 ± 0.110	0.482 ± 0.096	11.699	<0.001	0.464	0.002
BRD-ABS	α	0.420 ± 0.108	0.440 ± 0.114	0.670 ± 0.120	14.834	<0.001	0.524	<0.001
GM-PM	β	0.520 ± 0.106	0.540 ± 0.100	0.781 ± 0.116	18.192	<0.001	0.574	<0.001
GM-PM	γ	0.157 ± 0.048	0.171 ± 0.042	0.320 ± 0.054	34.399	<0.001	0.718	<0.001
VM-LD	β	0.071 ± 0.042	0.079 ± 0.052	0.290 ± 0.041	75.185	<0.001	0.848	<0.001
BF-ABS	β	0.364 ± 0.106	0.482 ± 0.102	0.612 ± 0.116	13.129	<0.001	0.493	0.001

Note: Values are mean ± SD. AZ was calculated from temporally normalized, surrogate-corrected TFC maps and analyzed without Fisher Z transformation. *p* values represent three-group omnibus comparisons using one-way ANOVA (all mean-centered Levene *p* > 0.05); partial η^2^ indicates effect size. Benjamini–Hochberg correction was applied across all 63 forehand-drive outcomes (significance: q < 0.05). Complete results are provided in Supplementary Table S1, with post hoc comparisons in the Supplementary Materials. Group A: loaded back squat; Group B: low-intensity electrical stimulation plus loaded back squat; Group C: NMES plus loaded back squat.

**Table 3:** Between-group comparisons of intermuscular time–frequency coherence during the forehand slice.

Muscle Pair	Frequency Band	Group A	Group B	Group C	Test (df)	F	Raw *p*	Partial η^2^	FDR q
BB-LD	α	0.190 ± 0.080	0.210 ± 0.084	0.480 ± 0.075	F(2,27)	41.421	<0.001	0.754	<0.001
BRD-ABS	γ	0.170 ± 0.048	0.260 ± 0.056	0.453 ± 0.106	Welch F(2, 16.90)	30.16	<0.001	0.781	<0.001
GM-ABS	α	0.109 ± 0.047	0.194 ± 0.109	0.461 ± 0.102	Welch F(2, 15.42)	47.466	<0.001	0.86	<0.001
VM-ABS	α	0.120 ± 0.051	0.140 ± 0.055	0.479 ± 0.171	Welch F(2, 16.43)	19.618	<0.001	0.705	<0.001
GLM-ABS	β	0.261 ± 0.107	0.310 ± 0.109	0.576 ± 0.102	F(2,27)	25.667	<0.001	0.655	<0.001
TA-ABS	γ	0.181 ± 0.109	0.212 ± 0.119	0.515 ± 0.217	Welch F(2, 17.09)	9.384	0.002	0.523	0.019

Note: Values are mean ± SD. AZ was calculated from temporally normalized, surrogate-corrected TFC maps and analyzed without Fisher Z transformation. *p* values represent three-group omnibus comparisons using ordinary one-way ANOVA or Welch’s ANOVA, as indicated. Benjamini–Hochberg correction was applied across all 63 forehand-slice outcomes (significance: q < 0.05). Complete results are provided in Supplementary Table S2, with post hoc comparisons in the Supplementary Materials. Group A: loaded back squat; Group B: low-intensity electrical stimulation plus loaded back squat; Group C: NMES plus loaded back squat.

**Table 4:** Bonferroni-adjusted pairwise comparisons for FDR-significant AZ outcomes during the forehand drive.

Muscle Pair	Band	A vs. B: Adjusted *p*	A vs. C: Adjusted *p*	B vs. C: Adjusted *p*
BB–ABS	β	1.000	0.003	0.003
BB–ABS	γ	1.000	<0.001	0.001
BB–LD	α	1.000	<0.001	0.004
BRD–ABS	α	1.000	<0.001	0.001
GM–PM	β	1.000	<0.001	<0.001
GM–PM	γ	1.000	<0.001	<0.001
VM–LD	β	1.000	<0.001	<0.001
BF–ABS	β	0.061	<0.001	0.049

Note: Pairwise independent-samples *t* tests were performed for the eight outcomes that remained significant after FDR correction of the omnibus tests. Each comparison included 10 participants per group (df = 18). *p* values were Bonferroni-adjusted across the three pairwise comparisons within each outcome. Values are reported to three decimal places; values below 0.001 are reported as <0.001. Statistical significance was determined using unrounded adjusted *p* values, with adjusted *p* < 0.05 considered significant. Group A: loaded back squat; Group B: low-intensity electrical stimulation plus loaded back squat; Group C: NMES plus loaded back squat.

**Table 5:** Multiplicity-adjusted pairwise comparisons for FDR-significant AZ outcomes during the forehand slice.

Muscle Pair	Band	Pairwise Method	A vs. B: Adjusted *p*	A vs. C: Adjusted *p*	B vs. C: Adjusted *p*
BB–LD	α	Bonferroni	1.000	<0.001	<0.001
BRD–ABS	γ	Games–Howell	0.003	<0.001	<0.001
GM–ABS	α	Games–Howell	0.100	<0.001	<0.001
VM–ABS	α	Games–Howell	0.681	<0.001	<0.001
GLM–ABS	β	Bonferroni	0.956	<0.001	<0.001
TA–ABS	γ	Games–Howell	0.814	0.002	0.005

Note: Pairwise comparisons were performed for the six outcomes that remained significant after FDR correction of the omnibus tests. Each comparison included 10 participants per group. Bonferroni-adjusted independent-samples *t* tests were used for BB–LD α and GLM–ABS β; Games–Howell comparisons were used for the remaining four outcomes. *p* values are reported to three decimal places; values below 0.001 are reported as <0.001. Statistical significance was determined using unrounded adjusted *p* values, with adjusted *p* < 0.05 considered significant. Group A: loaded back squat; Group B: low-intensity electrical stimulation plus loaded back squat; Group C: NMES plus loaded back squat.

## Data Availability

The data supporting the findings of this study are available within the article and its Supplementary Materials.

## Supplementary Materials

The following supporting information can be downloaded at: https://www.mdpi.com/article/10.3390/s26216902/s1, Table S1: Between-group comparisons of intermuscular time–frequency coherence during the forehand drive; Table S2: Between-group comparisons of intermuscular time–frequency coherence during the forehand slice.
