# Supplementary material for: Wearable Surface Electromyography Sensor-Based Assessment of Intermuscular Time–Frequency Coherence Following Neuromuscular Electrical Stimulation Combined with Loaded Back Squats During Tennis Forehand Strokes
Source: Sensors (Basel). 2026 Oct 31;26(21):6902. doi: 10.3390/s26216902 (PMC13766327; doi:10.3390/s26216902)
Supplement: Supplementary file 1 [file sensors-26-06902-s001.zip › Table S1.pdf]

Table S1. Between-group comparisons of intermuscular time–frequency coherence during the forehand drive

Muscle pair	Frequency band	Group A	Group B	Group C	F(2,27)	Raw P	Partial η²	FDR q
BB-ABS	α	0.100 ± 0.060	0.110 ± 0.066	0.165 ± 0.100	2.055	0.148	0.132	0.716
	**β**	**0.360 ± 0.149**	**0.390 ± 0.118**	**0.620 ± 0.143**	**10.747**	**<0.001**	**0.443**	**0.003**
	**γ**	**0.370 ± 0.177**	**0.410 ± 0.161**	**0.710 ± 0.145**	**13.231**	**<0.001**	**0.495**	**0.001**
**BB-LD**	**α**	**0.277 ± 0.102**	**0.306 ± 0.110**	**0.482 ± 0.096**	**11.699**	**<0.001**	**0.464**	**0.002**
	β	0.060 ± 0.036	0.066 ± 0.039	0.055 ± 0.032	0.223	0.802	0.016	0.999
	γ	0.045 ± 0.027	0.060 ± 0.037	0.059 ± 0.036	0.621	0.545	0.044	0.999
BB-PM	α	0.055 ± 0.034	0.064 ± 0.039	0.055 ± 0.032	0.203	0.818	0.015	0.999
	β	0.050 ± 0.031	0.060 ± 0.036	0.064 ± 0.039	0.448	0.643	0.032	0.999
	γ	0.059 ± 0.037	0.055 ± 0.034	0.065 ± 0.040	0.181	0.835	0.013	0.999
**BRD-ABS**	**α**	**0.420 ± 0.108**	**0.440 ± 0.114**	**0.670 ± 0.120**	**14.834**	**<0.001**	**0.524**	**<0.001**
	β	0.477 ± 0.165	0.426 ± 0.171	0.474 ± 0.177	0.277	0.760	0.02	0.999
	γ	0.051 ± 0.031	0.061 ± 0.038	0.050 ± 0.031	0.33	0.722	0.024	0.999
BRD-LD	α	0.061 ± 0.035	0.055 ± 0.034	0.060 ± 0.036	0.103	0.902	0.008	0.999
	β	0.055 ± 0.033	0.060 ± 0.036	0.056 ± 0.033	0.062	0.940	0.005	0.999
	γ	0.202 ± 0.118	0.169 ± 0.100	0.336 ± 0.178	4.224	0.025	0.238	0.177
BRD-PM	α	0.049 ± 0.031	0.065 ± 0.039	0.061 ± 0.038	0.541	0.588	0.039	0.999
	β	0.055 ± 0.032	0.055 ± 0.034	0.067 ± 0.043	0.384	0.685	0.028	0.999
	γ	0.045 ± 0.027	0.059 ± 0.036	0.066 ± 0.038	0.985	0.387	0.068	0.999
GM-ABS	α	0.658 ± 0.167	0.681 ± 0.177	0.679 ± 0.156	0.057	0.945	0.004	0.999
	β	0.611 ± 0.237	0.641 ± 0.164	0.649 ± 0.172	0.106	0.900	0.008	0.999
	γ	0.530 ± 0.103	0.520 ± 0.171	0.521 ± 0.176	0.013	0.987	0.001	0.999
GM-LD	α	0.316 ± 0.161	0.410 ± 0.166	0.519 ± 0.175	3.675	0.039	0.214	0.244
	β	0.360 ± 0.168	0.457 ± 0.160	0.548 ± 0.184	3.029	0.065	0.183	0.373
	γ	0.154 ± 0.091	0.151 ± 0.091	0.167 ± 0.103	0.084	0.920	0.006	0.999
GM-PM	α	0.056 ± 0.034	0.060 ± 0.037	0.050 ± 0.030	0.233	0.794	0.017	0.999
	**β**	**0.520 ± 0.106**	**0.540 ± 0.100**	**0.781 ± 0.116**	**18.192**	**<0.001**	**0.574**	**<0.001**
	**γ**	**0.157 ± 0.048**	**0.171 ± 0.042**	**0.320 ± 0.054**	**34.399**	**<0.001**	**0.718**	**<0.001**
VM-ABS	α	0.059 ± 0.035	0.065 ± 0.039	0.059 ± 0.038	0.086	0.918	0.006	0.999
	β	0.050 ± 0.032	0.060 ± 0.035	0.070 ± 0.044	0.705	0.503	0.05	0.999
	γ	0.066 ± 0.040	0.061 ± 0.036	0.070 ± 0.043	0.147	0.864	0.011	0.999
VM-LD	α	0.055 ± 0.034	0.055 ± 0.032	0.064 ± 0.040	0.238	0.790	0.017	0.999
	**β**	**0.071 ± 0.042**	**0.079 ± 0.052**	**0.290 ± 0.041**	**75.185**	**<0.001**	**0.848**	**<0.001**

	γ	0.054 ± 0.033	0.060 ± 0.037	0.065 ± 0.039	0.24	0.788	0.017	0.999
VM-PM	α	0.064 ± 0.039	0.060 ± 0.037	0.071 ± 0.042	0.194	0.825	0.014	0.999
	β	0.060 ± 0.037	0.068 ± 0.043	0.066 ± 0.040	0.118	0.889	0.009	0.999
	γ	0.054 ± 0.033	0.064 ± 0.040	0.075 ± 0.046	0.693	0.509	0.049	0.999
BF-ABS	α	0.283 ± 0.172	0.360 ± 0.170	0.427 ± 0.227	1.427	0.258	0.096	0.954
	**β**	**0.364 ± 0.106**	**0.482 ± 0.102**	**0.612 ± 0.116**	**13.129**	**<0.001**	**0.493**	**0.001**
	γ	0.059 ± 0.036	0.056 ± 0.034	0.065 ± 0.040	0.164	0.850	0.012	0.999
BF-LD	α	0.060 ± 0.035	0.060 ± 0.037	0.060 ± 0.037	0.002	0.998	0	0.999
	β	0.050 ± 0.030	0.064 ± 0.041	0.065 ± 0.041	0.522	0.599	0.037	0.999
	γ	0.056 ± 0.033	0.056 ± 0.033	0.071 ± 0.043	0.537	0.591	0.038	0.999
BF-PM	α	0.065 ± 0.039	0.065 ± 0.039	0.065 ± 0.039	0.001	0.999	0	0.999
	β	0.379 ± 0.175	0.472 ± 0.173	0.518 ± 0.165	1.714	0.199	0.113	0.784
	γ	0.056 ± 0.034	0.070 ± 0.041	0.069 ± 0.043	0.382	0.686	0.028	0.999
GLM-ABS	α	0.060 ± 0.037	0.070 ± 0.041	0.064 ± 0.038	0.194	0.825	0.014	0.999
	β	0.270 ± 0.111	0.258 ± 0.115	0.261 ± 0.100	0.033	0.968	0.002	0.999
	γ	0.395 ± 0.100	0.485 ± 0.164	0.492 ± 0.113	1.747	0.193	0.115	0.784
GLM-LD	α	0.055 ± 0.033	0.068 ± 0.042	0.071 ± 0.041	0.47	0.630	0.034	0.999
	β	0.061 ± 0.036	0.060 ± 0.036	0.076 ± 0.046	0.522	0.599	0.037	0.999
	γ	0.065 ± 0.040	0.064 ± 0.040	0.064 ± 0.039	0.004	0.996	0	0.999
GLM-PM	α	0.060 ± 0.036	0.064 ± 0.039	0.054 ± 0.033	0.197	0.822	0.014	0.999
	β	0.060 ± 0.037	0.060 ± 0.036	0.070 ± 0.042	0.263	0.771	0.019	0.999
	γ	0.383 ± 0.238	0.479 ± 0.230	0.570 ± 0.167	1.915	0.167	0.124	0.751
TA-ABS	α	0.391 ± 0.244	0.402 ± 0.237	0.503 ± 0.294	0.571	0.572	0.041	0.999
	β	0.339 ± 0.166	0.436 ± 0.233	0.534 ± 0.228	2.145	0.137	0.137	0.716
	γ	0.366 ± 0.219	0.450 ± 0.168	0.489 ± 0.293	0.732	0.490	0.051	0.999
TA-LD	α	0.055 ± 0.033	0.066 ± 0.040	0.060 ± 0.037	0.222	0.803	0.016	0.999
	β	0.055 ± 0.033	0.060 ± 0.037	0.070 ± 0.043	0.387	0.683	0.028	0.999
	γ	0.064 ± 0.038	0.060 ± 0.037	0.071 ± 0.043	0.194	0.825	0.014	0.999
TA-PM	α	0.058 ± 0.035	0.071 ± 0.041	0.065 ± 0.040	0.286	0.753	0.021	0.999
	β	0.054 ± 0.034	0.065 ± 0.039	0.076 ± 0.046	0.767	0.474	0.054	0.999
	γ	0.064 ± 0.039	0.061 ± 0.036	0.069 ± 0.042	0.12	0.887	0.009	0.999

Note: Values are presented as mean ± standard deviation. Values are presented as mean ± standard deviation. AZ was calculated from the temporally normalized, surrogate-corrected TFC map within each predefined frequency band. The participant-level AZ values were entered directly into the statistical analysis without Fisher Z transformation. Between-group differences were examined using one-way analysis of variance because all outcomes satisfied the homogeneity-of-variance assumption according

to the mean-centered Levene test (all $P > 0.05$). Partial η^2 represents the effect size. Raw P values from all 63 muscle-pair × frequency-band omnibus tests for the forehand drive were adjusted together using the Benjamini–Hochberg false discovery rate procedure. An FDR-adjusted q value < 0.05 was considered statistically significant. Each p-value represents an omnibus comparison among the three groups for the corresponding muscle-pair × frequency-band outcome, using one-way ANOVA or Welch's ANOVA, as appropriate. The q-values are the corresponding Benjamini–Hochberg FDR-adjusted p-values. A significant omnibus result indicates that at least one group differs from another; the specific group differences are identified by the post hoc comparisons reported in the supplementary materials. Group A performed loaded back squats alone; Group B received low-intensity electrical stimulation without visible muscle contractions during the same squat protocol; and Group C received NMES sufficient to elicit visible muscle contractions during the same squat protocol.
