# Supplementary material for: Wearable Surface Electromyography Sensor-Based Assessment of Intermuscular Time–Frequency Coherence Following Neuromuscular Electrical Stimulation Combined with Loaded Back Squats During Tennis Forehand Strokes
Source: Sensors (Basel). 2026 Oct 31;26(21):6902. doi: 10.3390/s26216902 (PMC13766327; doi:10.3390/s26216902)
Supplement: Supplementary file 1 [file sensors-26-06902-s001.zip › Table S2.pdf]

Table S2. Between-group comparisons of intermuscular time–frequency coherence during the forehand slice

Muscle pair	Frequency band	Group A	Group B	Group C	Test (df)	F	Raw P	Partial η²	FDR q
BB-ABS	α	0.156 ± 0.096	0.238 ± 0.110	0.252 ± 0.101	F(2, 27)	2.54	0.098	0.158	0.683
	β	0.130 ± 0.048	0.141 ± 0.054	0.151 ± 0.044	F(2, 27)	0.447	0.644	0.032	0.965
	γ	0.054 ± 0.034	0.064 ± 0.038	0.060 ± 0.036	F(2, 27)	0.185	0.832	0.014	0.965
**BB-LD**	**α**	**0.190 ± 0.080**	**0.210 ± 0.084**	**0.480 ± 0.075**	**F(2, 27)**	**41.421**	**<0.001**	**0.754**	**<0.001**
	β	0.067 ± 0.040	0.071 ± 0.044	0.064 ± 0.039	F(2, 27)	0.06	0.942	0.004	0.965
	γ	0.049 ± 0.031	0.057 ± 0.033	0.049 ± 0.031	F(2, 27)	0.191	0.827	0.014	0.965
BB-PM	α	0.391 ± 0.233	0.483 ± 0.218	0.573 ± 0.175	F(2, 27)	1.865	0.174	0.121	0.965
	β	0.416 ± 0.252	0.455 ± 0.179	0.551 ± 0.227	F(2, 27)	0.993	0.384	0.068	0.965
	γ	0.416 ± 0.168	0.497 ± 0.177	0.587 ± 0.237	F(2, 27)	1.886	0.171	0.123	0.965
BRD-ABS	α	0.055 ± 0.033	0.064 ± 0.040	0.054 ± 0.033	F(2, 27)	0.255	0.777	0.019	0.965
	β	0.055 ± 0.034	0.065 ± 0.039	0.059 ± 0.036	F(2, 27)	0.161	0.852	0.012	0.965
	**γ**	**0.170 ± 0.048**	**0.260 ± 0.056**	**0.453 ± 0.106**	**Welch F(2, 16.90)**	**30.16**	**<0.001**	**0.781**	**<0.001**
BRD-LD	α	0.191 ± 0.106	0.188 ± 0.111	0.221 ± 0.129	F(2, 27)	0.251	0.780	0.018	0.965
	β	0.055 ± 0.033	0.064 ± 0.039	0.060 ± 0.037	F(2, 27)	0.154	0.858	0.011	0.965
	γ	0.056 ± 0.033	0.066 ± 0.040	0.059 ± 0.037	F(2, 27)	0.212	0.810	0.015	0.965
BRD-PM	α	0.064 ± 0.040	0.076 ± 0.045	0.066 ± 0.040	F(2, 27)	0.241	0.787	0.018	0.965
	β	0.084 ± 0.053	0.079 ± 0.042	0.089 ± 0.048	F(2, 27)	0.119	0.888	0.009	0.965
	γ	0.383 ± 0.110	0.411 ± 0.054	0.419 ± 0.051	Welch F(2, 16.96)	0.433	0.655	0.049	0.965
**GM-ABS**	**α**	**0.109 ± 0.047**	**0.194 ± 0.109**	**0.461 ± 0.102**	**Welch F(2, 15.42)**	**47.466**	**<0.001**	**0.86**	**<0.001**
	β	0.052 ± 0.030	0.056 ± 0.033	0.055 ± 0.034	F(2, 27)	0.038	0.962	0.003	0.965

	γ	0.046 ± 0.028	0.065 ± 0.040	0.065 ± 0.041	F(2, 27)	0.915	0.413	0.063	0.965
GM-LD	α	0.055 ± 0.032	0.059 ± 0.035	0.073 ± 0.047	F(2, 27)	0.652	0.529	0.046	0.965
	β	0.059 ± 0.036	0.059 ± 0.035	0.076 ± 0.047	F(2, 27)	0.587	0.563	0.042	0.965
	γ	0.054 ± 0.034	0.065 ± 0.039	0.060 ± 0.035	F(2, 27)	0.244	0.785	0.018	0.965
GM-PM	α	0.212 ± 0.047	0.221 ± 0.054	0.230 ± 0.044	F(2, 27)	0.342	0.713	0.025	0.965
	β	0.060 ± 0.037	0.071 ± 0.041	0.064 ± 0.040	F(2, 27)	0.187	0.830	0.014	0.965
	γ	0.059 ± 0.036	0.070 ± 0.044	0.065 ± 0.039	F(2, 27)	0.197	0.823	0.014	0.965
**VM-ABS**	**α**	**0.120 ± 0.051**	**0.140 ± 0.055**	**0.479 ± 0.171**	**Welch F(2, 16.43)**	**19.618**	**<0.001**	**0.705**	**<0.001**
	β	0.055 ± 0.034	0.056 ± 0.035	0.050 ± 0.031	F(2, 27)	0.094	0.911	0.007	0.965
	γ	0.478 ± 0.224	0.565 ± 0.167	0.553 ± 0.292	F(2, 27)	0.406	0.670	0.029	0.965
VM-LD	α	0.060 ± 0.036	0.068 ± 0.041	0.064 ± 0.039	F(2, 27)	0.116	0.891	0.009	0.965
	β	0.060 ± 0.036	0.071 ± 0.042	0.064 ± 0.040	F(2, 27)	0.182	0.834	0.013	0.965
	γ	0.061 ± 0.035	0.070 ± 0.042	0.065 ± 0.040	F(2, 27)	0.121	0.886	0.009	0.965
VM-PM	α	0.062 ± 0.036	0.070 ± 0.043	0.070 ± 0.043	F(2, 27)	0.138	0.872	0.01	0.965
	β	0.065 ± 0.040	0.073 ± 0.046	0.073 ± 0.043	F(2, 27)	0.104	0.901	0.008	0.965
	γ	0.209 ± 0.110	0.251 ± 0.146	0.230 ± 0.103	F(2, 27)	0.311	0.735	0.023	0.965
BF-ABS	α	0.055 ± 0.033	0.065 ± 0.039	0.061 ± 0.036	F(2, 27)	0.18	0.836	0.013	0.965
	β	0.055 ± 0.034	0.065 ± 0.041	0.065 ± 0.039	F(2, 27)	0.212	0.810	0.015	0.965
	γ	0.055 ± 0.033	0.064 ± 0.040	0.060 ± 0.035	F(2, 27)	0.136	0.874	0.01	0.965
BF-LD	α	0.059 ± 0.038	0.071 ± 0.042	0.065 ± 0.041	F(2, 27)	0.204	0.817	0.015	0.965
	β	0.061 ± 0.036	0.061 ± 0.037	0.077 ± 0.047	F(2, 27)	0.528	0.596	0.038	0.965
	γ	0.059 ± 0.036	0.060 ± 0.036	0.076 ± 0.045	F(2, 27)	0.558	0.579	0.04	0.965

BF-PM	α	0.454 ± 0.271	0.542 ± 0.277	0.643 ± 0.230	F(2, 27)	1.327	0.282	0.089	0.965
	β	0.060 ± 0.038	0.077 ± 0.046	0.070 ± 0.040	F(2, 27)	0.411	0.667	0.03	0.965
	γ	0.396 ± 0.168	0.409 ± 0.107	0.424 ± 0.158	F(2, 27)	0.092	0.912	0.007	0.965
GLM-ABS	α	0.065 ± 0.039	0.071 ± 0.042	0.065 ± 0.040	F(2, 27)	0.068	0.935	0.005	0.965
	**β**	**0.261 ± 0.107**	**0.310 ± 0.109**	**0.576 ± 0.102**	**F(2, 27)**	**25.667**	**<0.001**	**0.655**	**<0.001**
	γ	0.049 ± 0.031	0.061 ± 0.036	0.055 ± 0.033	F(2, 27)	0.301	0.743	0.022	0.965
GLM-LD	α	0.060 ± 0.036	0.065 ± 0.040	0.058 ± 0.037	F(2, 27)	0.074	0.929	0.005	0.965
	β	0.061 ± 0.035	0.061 ± 0.036	0.055 ± 0.032	F(2, 27)	0.119	0.888	0.009	0.965
	γ	0.055 ± 0.034	0.066 ± 0.040	0.054 ± 0.034	F(2, 27)	0.326	0.724	0.024	0.965
GLM-PM	α	0.051 ± 0.030	0.056 ± 0.033	0.049 ± 0.031	F(2, 27)	0.122	0.886	0.009	0.965
	β	0.070 ± 0.042	0.065 ± 0.041	0.075 ± 0.044	F(2, 27)	0.144	0.866	0.011	0.965
	γ	0.580 ± 0.108	0.560 ± 0.103	0.613 ± 0.113	F(2, 27)	0.607	0.552	0.043	0.965
TA-ABS	α	0.312 ± 0.194	0.428 ± 0.242	0.382 ± 0.170	F(2, 27)	0.816	0.453	0.057	0.965
	β	0.055 ± 0.034	0.062 ± 0.035	0.050 ± 0.030	F(2, 27)	0.35	0.708	0.025	0.965
	γ	**0.181 ± 0.109**	**0.212 ± 0.119**	**0.515 ± 0.217**	**Welch F(2, 17.09)**	**9.384**	**0.002**	**0.523**	**0.019**
TA-LD	α	0.452 ± 0.106	0.479 ± 0.115	0.611 ± 0.105	F(2, 27)	6.097	0.007	0.311	0.059
	β	0.060 ± 0.037	0.064 ± 0.041	0.061 ± 0.036	F(2, 27)	0.036	0.965	0.003	0.965
	γ	0.072 ± 0.043	0.071 ± 0.042	0.066 ± 0.039	F(2, 27)	0.061	0.941	0.004	0.965
TA-PM	α	0.061 ± 0.037	0.059 ± 0.036	0.075 ± 0.047	F(2, 27)	0.461	0.635	0.033	0.965
	β	0.060 ± 0.037	0.068 ± 0.043	0.066 ± 0.040	F(2, 27)	0.104	0.901	0.008	0.965
	γ	0.376 ± 0.236	0.486 ± 0.217	0.588 ± 0.170	F(2, 27)	2.577	0.095	0.16	0.683

Note: Values are presented as mean ± standard deviation. Values are presented as mean ± standard deviation. AZ was calculated from the temporally normalized, surrogate-corrected TFC map within each predefined frequency

band. The participant-level AZ values were entered directly into the statistical analysis without Fisher Z transformation. Homogeneity of variance was evaluated separately for each outcome using the mean-centered Levene test. Ordinary one-way analysis of variance was used when Levene's $P \geq 0.05$, whereas Welch's analysis of variance was used when Levene's $P < 0.05$. Accordingly, the denominator degrees of freedom for Welch's F tests were estimated using the Welch–Satterthwaite method and may be non-integer. Partial η^2 was derived from the reported F statistic and its corresponding degrees of freedom. Raw P values from all 63 muscle-pair × frequency-band omnibus tests for the forehand slice were adjusted together using the Benjamini–Hochberg false discovery rate procedure. An FDR-adjusted q value < 0.05 was considered statistically significant. For outcomes with $q < 0.05$, Bonferroni-adjusted pairwise comparisons were used following ordinary ANOVA, whereas Games–Howell pairwise comparisons were used following Welch's ANOVA. ^a^P < 0.05 versus the loaded back squat group; ^b^P < 0.05 versus the sham NMES + loaded back squat group. NMES, neuromuscular electrical stimulation; FDR, false discovery rate. Each p-value represents an omnibus comparison among the three groups for the corresponding muscle-pair × frequency-band outcome, using one-way ANOVA or Welch's ANOVA, as appropriate. The q-values are the corresponding Benjamini–Hochberg FDR-adjusted p-values. A significant omnibus result indicates that at least one group differs from another; the specific group differences are identified by the post hoc comparisons reported in the supplementary materials.

Group A performed loaded back squats alone; Group B received low-intensity electrical stimulation without visible muscle contractions during the same squat protocol; and Group C received NMES sufficient to elicit visible muscle contractions during the same squat protocol.
